# Age- and glycemia-related miR-126-3p levels in plasma and endothelial cells

**DOI:** 10.18632/aging.100693

**Published:** 2014-10-07

**Authors:** Fabiola Olivieri, Massimiliano Bonafè, Liana Spazzafumo, Mirko Gobbi, Francesco Prattichizzo, Rina Recchioni, Fiorella Marcheselli, Lucia La Sala, Roberta Galeazzi, Maria Rita Rippo, Gianluca Fulgenzi, Sabrina Angelini, Raffaella Lazzarini, Anna Rita Bonfigli, Francesca Brugè, Luca Tiano, Stefano Genovese, Antonio Ceriello, Massimo Boemi, Claudio Franceschi, Antonio Domenico Procopio, Roberto Testa

**Affiliations:** ^1^ Department of Clinical and Molecular Sciences, DISCLIMO, Università Politecnica delle Marche, Ancona, Italy; ^2^ Center of Clinical Pathology and Innovative Therapy, National Institute INRCA-IRCCS, Ancona, Italy; ^3^ Department of Experimental, Diagnostic and Specialty Medicine, DIMES, University of Bologna, Bologna, Italy; ^4^ CNR, National Research Council of Italy, Institute for Molecular Genetics, Unit of Bologna IOR, Bologna, Italy; ^5^ Laboratory of Musculoskeletal Cell Biology, IOR, Bologna, Italy; ^6^ Center of Biostatistics, INRCA-IRCCS National Institute, Ancona, Italy; ^7^ Institut d'Investigacions Biomèdiques August Pi i Sunyer (IDIBAPS), Barcelona, Spain; ^8^ Centro de Investigación Biomédica en Red de Diabetes y Enfermedades Metabólicas Asociadas (CIBERDEM), Barcelona, Spain; ^9^ Clinical & Molecular Diagnostic Laboratory, INRCA-IRCCS National Institute, Ancona, Italy; ^10^ Department of Pharmacy and Biotechnology, University of Bologna, Bologna, Italy; ^11^ Metabolic Diseases and Diabetology Unit, INRCA-IRCCS National Institute, Ancona, Italy; ^12^ Department of Dentistry and Clinical Sciences, Università Politecnica delle Marche, Ancona, Italy; ^13^ Department of Cardiovascular and Metabolic Diseases, IRCCS Gruppo Multimedica Sesto San Giovanni, Italy; ^14^ C.I.G. Interdepartmental Center "L. Galvani", University of Bologna, Bologna, Italy; ^15^ Experimental Models in Clinical Pathology, INRCA-IRCCS National Institute, Ancona, Italy

**Keywords:** miR-126-3p, T2DM, HUVEC, senescence

## Abstract

Circulating miR-126-3p levels were determined in 136 healthy subjects (CTRs) aged 20-90 years and 193 patients with type-2 diabetes mellitus (T2DMs) aged 40-80 years, to explore the combined effect of age and glycemic state on miR-126-3p expression. Moreover, intra/extracellular miR-126-3p levels were measured in human endothelial cells (HUVECs) undergoing senescence under normo/hyper-glycemic conditions.

Plasma miR-126-3p was significantly higher in the oldest compared with the youngest CTRs (<45 vs. >75 years; relative expression: 0.27±0.29 vs. 0.48±0.39, p=0.047). Age-based comparison between CTRs and T2DM demonstrated significantly different miR-126-3p levels only in the oldest (0.48±0.39 vs. 0.22±0.23, p<0.005). After multiple adjustments, miR-126-3p levels were seen to be lower in patients with poor glycemic control, compared with age-matched CTRs.

The age-related increase in plasma miR-126-3p found in CTRs was paralleled by a 5/6-fold increase in intra/extracellular miR-126-3p in *in vitro*-cultured HUVECs undergoing senescence. Notably, significant down-regulation of SPRED-1 protein, a validated miR-126-3p target, was found in senescent HUVECs. Moreover, miR-126-3p expression was down-regulated in intermediate-age HUVECs grown in high-glucose medium until senescence.

Aging/senescence-associated miR-126-3p up-regulation is likely a senescence-associated compensatory mechanism that is blunted when endothelial cells are exposed to high glucose levels, a phenomenon that probably occurs *in vivo* in T2DM patients.

## INTRODUCTION

MicroRNAs (miRNAs) are a broad class of small non-coding RNAs that act mainly by repressing gene expression through interactions with complementary sequences in coding and non-coding regions of mRNA targets [1]. In addition to such intracellular function, they can be secreted or released by cells within small membranous vesicles (e.g. exosomes, microparticles and apoptotic bodies) or packaged in high-density lipoprotein (HDL) or RNA-binding proteins (e.g. Argonaute) [2, 3, 4, 5], thus circulating in the blood-stream in a remarkably stable form [6, 7]. However, the origin of circulating miRNAs is in most instances unclear. Recently, senescent cells have emerged as a possible source of circulating miRNAs [8]. “Cell senescence” is a specific phenotype characterized by an irreversible growth arrest associated with dramatic changes in cell morphology, structure, and functions [9]. A phenotype closely related to the senescence phenotype is the “senescence-associated secretory phenotype” (SASP). Albeit in growth arrest, senescent cells remain metabolically active, secreting several different bioactive molecules that contribute to the creation of a pro-inflammatory microenvironment [10].

Since accumulation of senescent cells in the elderly seems to be a detrimental factor in aging and age-related disorders [11], and differences in circulating miRNAs have been detected in a variety of age-associated diseases [12-15], it is conceivable that the conditions interfering with miRNA synthesis and/or release are related both to aging and disease processes. Interestingly, comparison of the miRNAs involved in cellular senescence with those involved in age-associated diseases showed that these *in vitro* and *in vivo* conditions share several miRNAs [16-18]. Indisputably, the discovery of circulating miRNAs has opened a new era in the field of systemic and tissue-specific diagnostic/prognostic biomarkers for a number of age-related diseases, such as type 2 diabetes mellitus (T2DM), acute myocardial infarction (AMI), congestive heart failure (CHF), and cancer [19-25]. Even though some studies have addressed possible changes in circulating miRNA levels during normal aging [8,26,27], none have analyzed age-related circulating miRNA levels in the presence of disease. Notably, a range of biological variables can accelerate the senescence rate *in vivo* and *in vitro*; among them glucose plays an important role [28, 29]. High glucose levels induce earlier senescence, while glucose restriction inhibits cell senescence and significantly extends cellular lifespan, probably by modulating the epigenetic control of gene expression [29-31]. Thus, the cell senescence status and glucose levels may be the master modulators of miRNA release in the bloodstream, and the identification of miRNAs modulated by age and/or glycemic conditions could be a useful approach to identifying novel diagnostic and therapeutic strategies. The human miRNA (miR)-126 has been extensively studied in plasma and circulating cells, because its expression is very high in endothelial cells (ECs), endothelial progenitor cells (EPCs) and platelets [6, 32-33]. MiR-126 plays a pivotal role in modulating vascular development and homeostasis, targeting specific mRNAs such as CXCL12, VCAM-1, SPRED-1 and PIK3R2, thus contributing to the endothelial dysfunction associated with the development of diabetes and its complications [34-37]. A number of papers suggest that circulating miR-126 acts as an intercellular messenger mainly released by ECs and internalized primarily by monocytes and smooth muscle cells [34, 38-39]. Its transfer modulation may be an important strategy to prevent or delay endothelial dysfunction [36, 40-41]. Notably, a significant increase in circulating miR-126 has been measured in patients with age-related diseases such as AMI and stable as well as unstable angina [21, 22, 41].

Yet, significant miR-126 down-regulation has been described in plasma and circulating cells of T2DM patients [22, 38, 40], of CHF patients [21, 42], and in different cancers [43]. It is therefore conceivable that different stimuli, inducing different cellular fates in different cell types, modulate circulating miR-126. Since senescent cells have been widely studied as a model of aging and cells cultured *in vitro* under hyperglycemic conditions can be used as models of diabetes, we investigated age-related changes in miR-126 levels in healthy subjects and in T2DM patients and explored senescence–associated and hyper-glycemia-associated changes in an *in vitro* model of human ECs, i.e. human umbilical vein endothelial cells (HUVECs).

## RESULTS

### Plasma miR-126-3p levels in healthy subjects of different ages

To identify age-related changes in miR-126-3p in CTR subjects, participants were subdivided into young (20-45 years, n=44 ), elderly (46-75 years, n=57) and old (≥ 75 years, n=35). The values of their biochemical variables are reported in Table 1.

**Table 1:** Biochemical variables of 136 healthy control subjects (CTR) divided into three age-groups

Variables	<45 yrs n=44	46-74 yrs n=57	≥75 yrs n=35	p value ●
**BMI**, *kg/m^2^*	**25.6 ± 4.9**	**27.2 ± 4.4**	**27.0 ± 6.1**	0.013
**Total cholesterol**, *mg/dL*	**194.3 ± 34.2**	**219.1 ± 36.4**	**213.8 ± 38.0**	<0.01
**HDL cholesterol**, *mg/dL*	**58.6 ± 13.3**	**58.4 ± 14.9**	**52.5 ± 13.7**	0.013
**Triglycerides**, *mmol/L*	**81.9 ± 55.7**	**105.8 ± 74.2**	**116.1 ± 44.2**	<0.01
**Glucose**, *mg/dL*	**90.2 ± 8.7**	**93.5 ± 10.0**	**96.1 ± 10.8**	<0.01
**HbA1c**, *%*	**5.5 ± 0.4**	5.7 ± 0.4	5.7 ± 0.4	**<0.01**
**Insulin**, *mcU/mL*	**5.9 ± 4.0**	**6.5 ± 5.0**	**8.7 ± 8.0**	<0.01
**WBC, 10^3^*/uL***	**6.6 ± 1.5**	**6.1 ± 1.6**	**5.9 ± 1.3**	0.021
**Platelets 10^3^*/uL***	**245.7 ± 57.2**	**228.2 ± 53.8**	**222.5 ± 63.5**	0.012
**PAI-1**, *ng/mL*	**19.1 ± 9.2**	**22.4 ± 11.8**	**25.7 ± 12.4**	<0.01
**Hs-CRP**, *mg/L*	**2.3 ± 3.4**	**3.7 ± 8.2**	**5.5 ± 11.2**	0.049
**Fibrinogen**, *mg/dL*	**264 ± 69**	**298 ± 72**	**341 ± 116**	<0.01
Creatinine, *mg/dL*	**0.78 ± 0.17**	**0.80 ± 0.29**	**0.87 ± 0.29**	**0.145**
**ApoAI, *mg/dL***	**175.4 ± 34.5**	**179.9 ± 30.3**	**170.8 ± 30.4**	0.043
**ApoB,*mg/dL***	**89.8 ± 31.0**	**103.1 ± 26.8**	**103.2 ± 28.3**	<0.01
**MiR-126 *(rel. expression a.u.)***	**0.27 ± 0.29**	**0.31 ± 0.22**	**0.48 ± 0.39**	0.033
**Telomere length *(T/S, a.u.)***	**0.53 ± 0.20**	**0.47 ± 0.18**	**0.48 ± 0.18**	0.020

Variables are expressed as mean (SD).

∉ p from ANOVA. Parameters showing significant differences between groups are in bold.

BMI = body mass index; WBC = White blood cells; PAI-1 = Plasminogen activator inhibitor-1; ApoAI = Apolipoprotein-AI; ApoB = Apolipoprotein B; a.u. =arbitrary units.

**Figure 1:**
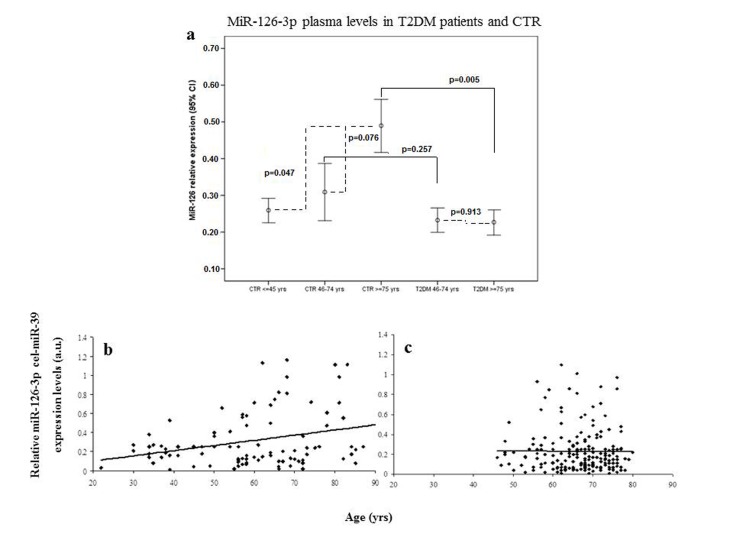
Relative miR-126-3p expression in plasma from 136 healthy subjects (CTR) and 193 patients (T2DM) divided into age groups Error bars (**a**) showing relative miR-126-3p expression (in a.u.) in plasma of young (20-45 years, n=44), elderly (46-74 years, n=57) and old (≥ 75 years, n=34) CTR and in plasma of T2DM subjects divided into two age groups (46-74 years, n=155 vs. ≥ 75 years, n=38). *p from t-test with Bonferroni correction for multiple comparisons. ANOVA, F= 4.887, p=0.001. Scatter plots (**b** and **c**) showing relative miR-126-3p expression (in arbitrary units, a.u.) according to age in CTR subjects (b) and T2DM patients (**c**). Data are expressed as mean of 2^ΔΔΔCt^ normalized with cel-miR-39.

Plasma miR-126-3p levels showed a significant age-related increase in the three subgroups (miR-126-3p levels expressed as arbitrary units [a.u.]: 0.27 ± 0.22, 0.31 ± 0.29 and 0.48 ± 0.39, respectively; Bonferroni t-test, young vs. old p=0.047 and elderly vs. old p=0.076; correlation coefficient R=0.10, p=0.004). ANOVA, F= 4.887, p=0.001. Error bars are reported in Fig. 1a, and scatter plots showing relative miR-126-3p expression (in arbitrary units, a.u.) in CTR and T2DM subjects were showed in Fig. 1b and 1c.

### Plasma miR-126-3p levels in T2DM patients of different ages

**Figure 2:**
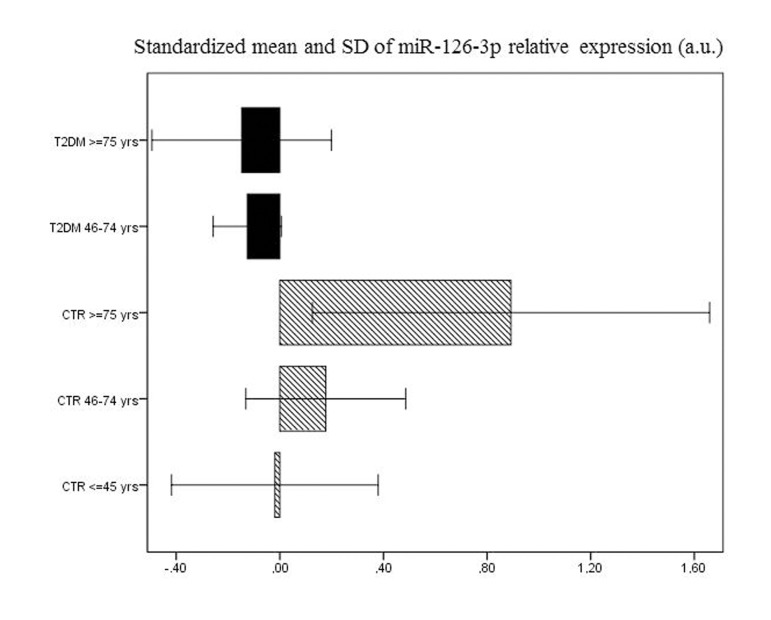
Relative miR-126-3p expression in 92 CTR and 193 T2DM subjects Standardized means of relative miR-126-3p expression (in a.u.) in 193 T2DM patients and 136 CTR subjects divided into age groups (T2DM: 46-74 years and ≥ 75 years; CTR: 20-45 years, 46-74 years and ≥ 75 years).

Age-related changes in miR-126-3p levels were further investigated in the 193 T2DM patients, divided into elderly (46-75 years, n=155) and old (≥ 75 years, n=38) patients. The values of the biochemical variables of the two groups are reported in Table 2. Age-related differences were not significant either in most of the biochemical variables or in plasma miR-126-3p (Fig. 1b and 1c). The standardized mean values of plasma miR-126-3p levels in patients and CTR subjects divided into age groups are reported in Figure 2. Standardized values offer an additional aid to data interpretation, since they provide the distance between mean values of relative miR-126-3p expression in the different age groups and the overall mean. The highest distance from the overall mean, about one standard deviation above the overall mean, was found in the miR-126-3p levels of the oldest CTR subjects (≥ 75 years, n=35).

**Table 2:** Biochemical variables of 193 patients with type 2 diabetes mellitus (T2DM) divided into two age groups

Variables	46-74 yrs n=155	≥75 yrs n=38	p value ●
**BMI**, *kg/m^2^*	**29.2 ± 4.6**	**27.6 ± 4.4**	<0.01
Total cholesterol, *mg/dL*	**208.0 ± 38.3**	**204.6 ± 36.3**	**0.456**
**HDL cholesterol**, *mg/dL*	**51.8 ± 14.7**	**56.5 ± 14.6**	<0.01
Triglycerides, *mmol/L*	**142.4 ± 112.4**	**130.0 ± 88.4**	**0.359**
Glucose, *mg/dL*	**164.5 ± 49.4**	**157.5 ± 43.7**	**0.245**
HbA1c, *%*	7.5 ± 1.3	7.4 ± 1.2	0.977
Insulin, *mcU/mL*	**7.4 ± 7.9**	**6.6 ± 6.8**	**0.407**
WBC, 10^3^*/uL*	**6.7 ± 1.7**	**6.7 ± 1.3**	**0.973**
Platelets 10^3^*/uL*	**217.0 ± 62.2**	**232.5 ± 104.0**	**0.069**
PAI-1, *ng/mL*	**21.3 ± 9.8**	**20.1 ± 9.7**	**0.352**
Hs-CRP, *mg/L*	**4.6 ± 7.1**	**4.2 ± 4.7**	**0.643**
Fibrinogen, *mg/dL*	**303 ± 78**	**310 ± 88**	**0.478.**
**Creatinine, *mg/dL***	**0.91 ± 0.29**	**1.03 ± 0.45**	<0.01
ApoAI, *mg/dL*	**166.6 ± 33.2**	**173.7 ± 385.3**	**0.087**
ApoB,*mg/dL*	**102.0 ± 26.5**	**100.5 ± 26.5**	**0.637**
MiR-126 *(rel. expression a.u.)*	**0.23 ± 0.22**	**0.22 ± 0.23**	**0.902**
**Telomere length *(T/S, a.u.)***	**0.45 ± 0.22**	**0.39 ± 0.56**	0.013

Variables are expressed as mean (SD)

∉ Independent sample t–test. Parameters showing significant differences between groups are in bold.

BMI = body mass index; WBC = White blood cells; PAI-1 =Plasminogen activator inhibitor-1; ApoAI = Apolipoprotein-AI; ApoB = Apolipoprotein B; a.u. =arbitrary unit.

Since the age range of the 193 T2DM patients was narrower than that of CTR subjects, a subset of 92 CTR subjects closely matching the T2DM group was used for subsequent comparisons. The respective biochemical and clinical variables are reported in Table 3. Comparisons of mean miR-126-3p levels showed significantly lower plasma levels in patients (t test=3.354, p=0.005). ANCOVA adjusted for the biochemical variables that were found to be significantly different between CTR and T2DM subjects by univariate analysis confirmed the significantly lower levels found in T2DM subjects (F test=10.964, p=0.001). Fasting glucose was excluded from the analysis because glycemia is a measure closely related to disease diagnosis. Subsequent analyses showed significant inverse correlations between fasting glucose, HbA1c, and miR-126-3p (R =-0.15, p=0.02; R=-0.15, p=0.01, respectively), whereas ApoAI and platelet number showed a significant positive correlation with circulating miR-126-3p levels (R=0.15, p=0.03, R=0.17, p<0.01, respectively). The partial correlation coefficients are reported in Supplementary Table 1 with a cut-off value of 0.15 and a p value <0.05. When the correlation between miR-126-3p and platelet counts was analyzed in T2DM patients it ceased to be significant (R=0.09, p>0.05), whereas the one between miR-126-3p and ApoA1 remained significant (R=0.16, p=0.02).

**Table 3:** Biochemical and clinical variables of healthy control (CTR) subjects and patients with type 2 diabetes mellitus (T2DM)

Variables	CTR n = 92	T2DM n = 193	p value ●
Age, *ys*	65.8 ± 11.6	65.9 ± 7.8	0.94
**BMI, *kg/m^2^***	26.8 ± 6.2	28.4 ± 3.7	**<0.01**
**Total cholesterol, *mg/dL***	218.3 ± 42.4	207.2 ± 39.1	**0.02**
**HDL cholesterol, *mg/dL***	59.5 ± 13.3	53.5 ± 16.7	**<0.01**
**Triglycerides, *mmol/L***	102.3 ± 64.1	144.6 ± 104.4	**<0.01**
**Glucose, *mg/dL***	93.2 ± 8.5	168.3 ± 50.8	**<0.01**
**HbA1c, *%***	5.7 ± 0.36	7.6 ± 1.2	**<0.01**
**Insulin, *mcU/mL***	5.24 ± 3.03	6.41 ± 3.56	**0.04**
**WBC, 10^3^*/uL***	6.1 ± 1.33	6.7 ± 1.51	**<0.01**
**Platelets 10^3^*/uL***	224.8 ± 52.2	210.7 ± 62.0	**0.05**
PAI-1, *ng/mL*	19.6 ± 10.79	20.52 ± 9.8	N.S.
**Hs-CRP, *mg/L***	2.5 ± 2.7	3.6 ± 4.1	**0.03**
Fibrinogen, *mg/dL*	295 ± 49	290 ± 81	N.S.
**Creatinine, *mg/dL***	0.83 ± 0.29	0.94 ± 0.35	**0.02**
**ApoAI, *mg/dL***	182.4 ± 34.1	166.9 ± 36.6	**<0.01**
ApoB,*mg/dL*	108.6 ± 28.2	103.1 ± 27.1	N.S.
**MiR-126 *(rel. expression a.u.)***	0.34 ± 0.31	0.23 ± 0.21	**<0.01**
**Telomere length *(T/S, a.u.)***	0.49 ± 0.20	0.43 ± 0.21	**0.04**
**Duration of diabetes, *yrs***	0	17 ± 12	**<0.01**
Males, *% (n)*	47 (51.2)	109 (56.5)	N.S.
**Use of statins, *n (%)***	13 (14.1)	41 ( 21.2)	**<0.01**
**Use of sulfonylurea, *n (%)***	0	108 (56.0)	**<0.01**
**Use of metformin, *n (%)***	0	76 (39.4)	**<0.01**
**Use of insulin, *n (%)***	0	45 (23.3)	**<0.01**
**Use of anti-aggregant, *n (%)***	2 (2.5)	40 ( 21.5)	**<0.01**
**Hypertension, *n (%)***	34 (37.2)	124 (64.2)	**<0.01**
**Hypercholesterolaemia, *n (%)***	9 (10.2)	51 (26.4)	**<0.01**
**Previous AMI, *n (%)***	0	34 (17.6)	**<0.01**

Normally distributed variables are expressed as mean (SD) and categorical variables as percentage (n).

∉ Independent sample t –test.

Parameters showing significant differences between CTR and T2DM are in bold.

AMI= Acute Myocardial Infarction; BMI = body mass index; WBC = White blood cells; PAI-1 =Plasminogen activator inhibitor-1; ApoAI = Apolipoprotein-AI; ApoB = Apolipoprotein B; a.u. =arbitrary units.

The positive correlation found between plasma miR-126-3p and ApoA1 levels prompted a further test, to establish whether the drugs that affect ApoAI levels, taken by some of these patients, also impact circulating miR-126-3p levels. Comparison of plasma miR-126-3p between patients taking/not taking statins after adjustment for age, gender, fasting glucose and ApoAI concentration showed significantly lower values in patients who were not on statins (miR-126-3p a.u.: 41 T2DM+ statins vs. 152 T2DM–statins, 0.29 ± 0.25 vs. 0.21 ± 0.20, p=0.02) (data not shown).

Treatment with sulfonylurea, metformin, insulin and anti-inflammatories in T2DM patients was not associated with significant plasma miR-126-3p modulation.

**Figure 3:**
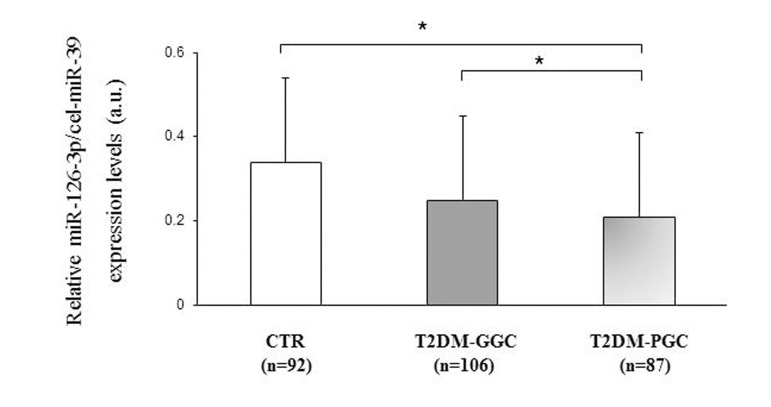
Relative miR-126-3p expression in 92 CTR and 193 T2DM subjects divided into those with good and poor glycemic control based on HbA1c levels Relative miR-126-3p expression (in arbitrary units, a.u.) in 92 CTR subjects and T2DM patients divided into those with good (GGC, n=106) and poor glycemic control (PGC, n=87) based on an HbA1c cut-off of 7% (53 mmol/mol) (a). Data are expressed as 2^ΔΔΔCt^ normalized with cel-miR-39. *GLM, adjustment for age, platelet count and ApoAI levels, p<0.05.

Furthermore, miR-126-3p expression was compared between patients divided into those with good (T2DM-GGC) and poor glycemic control (T2DM-PGC) based on an HbA1c cut-off of 7% (53 mmol/mol). T2DM-PGC patients (HbA1c ≥7 %, >53 mmol/mol) showed significantly lower miR-126-3p levels compared with T2DM-GGC subjects (HbA1c <7 %, <53 mmol/mol) (106 T2DM-PGC vs. 87 T2DM-GCC; miR-126-3p a.u.: 0.20±0.15 vs. 0.26±0.26, p=0.04) and with the subset of CTR participants (106 T2DM-PGC vs. 92 CTR; miR-126-3p a.u.: 0.20±0.17 vs 0.34±0.31 vs. p<0.01, Fig. 3), after adjustment for age, platelet count and ApoAI levels.

### MiR-126-3p levels in HUVECs undergoing senescence under normoglycemic conditions

To gain further insights into the contribution of endothelial cells to the systemic changes occurring in circulating miR-126-3p levels during aging, intra- and extracellular miR-126-3p levels were measured in *in vitro*-cultured HUVECs undergoing senescence.

**Figure 4:**
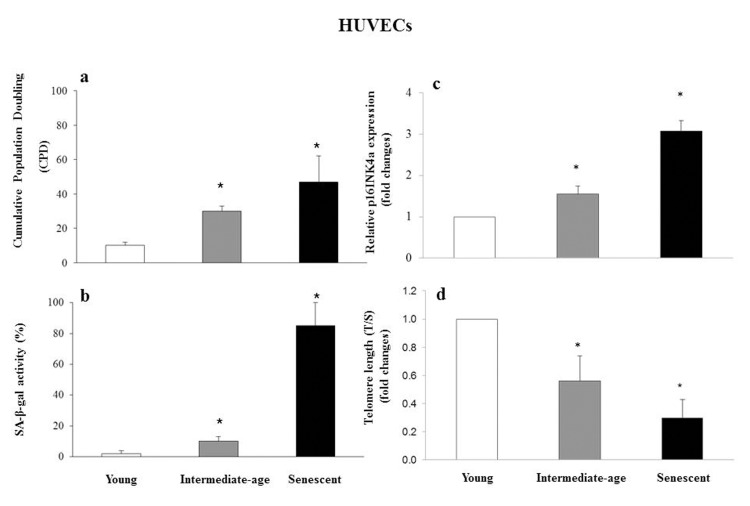
Characterization of young, intermediate-age and senescent HUVECs Bar chart showing cumulative population doubling (CPD) (**a**), SA-β-gal activity (%) (**b**), p16INK4a relative expression reported as fold changes vs. young cells (**c**) and telomere length (T/S) reported as fold changes vs. young cells (**d**), in young, intermediate age and senescent HUVECs. *GLM, p<0.05.

Senescence status was defined based on CPD (CPD= 40 ± 2 in senescent cells, CPD = 30 ± 2 in intermediate age cells, and CPD = 10 ± 2 in young cells) (Fig. 4a), SA-β-gal activity (85 ± 10 in senescent, 10 ± 4 in intermediate age and 2 ± 2 in young cells) (Fig. 4b), p16INK4a relative expression (fold increase: 3.07 ± 0.25 in senescent and 1.54 ± 0.22 in intermediate age vs. young cells) (Fig. 4c), and telomere length measured as T/S (fold decrease: 0.32 ± 0.13 in senescent and 0.59 ± 0.18 in intermediate-age vs. young cells) (Fig. 4d).

**Figure 5:**
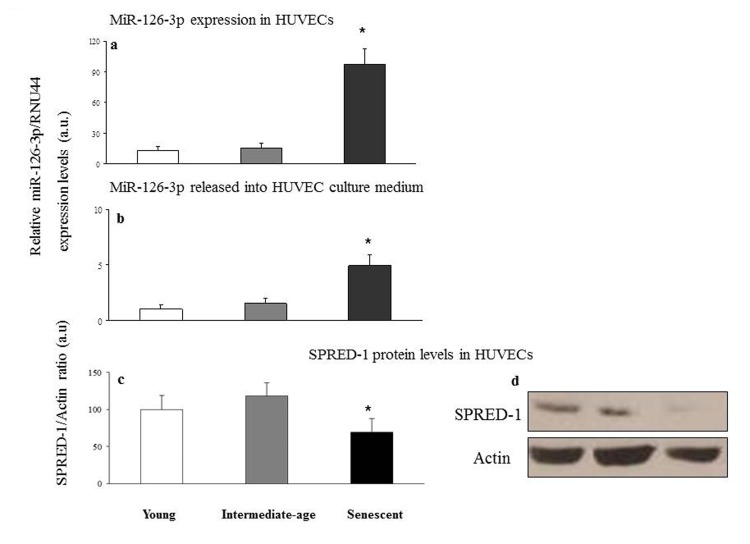
Relative miR-126-3p expression and SPRED-1 protein levels in young, intermediate age and senescent HUVECs Young, intermediate age and senescent HUVECs: intracellular miR-126-3p (**a**) and miR-126-3p recovered from conditioned medium (**b**). Data are expressed as 2^−ΔCt^ normalized with RNU44, and reported as arbitrary units (a.u.). Western blot and densitometric analysis of SPRED-1 (**c** and **d**, respectively) were performed in the same samples. Data from three independent experiments are expressed as percentage of intensity in young cells. *GLM, p<0.05.

A significant miR-126-3p increase was found in senescent compared with intermediate age and young cells (intracellular miR-126-3p: senescent vs. intermediate age cells, 97.8 ± 15 vs. 15.6 ± 5, p<0.05; senescent vs. young cells, 97.8 ± 15 vs. 12.9 ± 4, p<0.05; extracellular miR-126-3p: senescent vs. intermediate age cells, 4.9 ± 1.1 vs. 1.6 ± 0.6, p<0.05; senescent vs. young cells, 4.9 ± 1.1 vs. 1.1 ± 0.5, p<0.05) (Fig. 5a and 5b), with an approximately 6-fold and 5-fold increase in intra- and extracellular levels, respectively. Accordingly SPRED-1 protein, a validated target of miR-126-3p in HUVECs [36], was significantly down-regulated in senescent compared with intermediate age and young cells (Fig. 5c).

### MiR-126-3p levels in HUVECs undergoing senescence under hyperglycemic conditions

Since the *in vivo* effects of aging and glucose levels are extremely difficult to discriminate, we examined whether HUVECs exposed to high glucose concentrations until achievement of senescence affected miR-126-3p expression. Since cell senescence is defined as a state of irreversible growth arrest, we used intermediate-age HUVECs, which can still undergo replication, and cultured them in high-glucose medium (25 mM) until they reached replicative senescence as a model of the chronic hyperglycemic condition found *in vivo* in diabetic patients.

**Figure 6:**
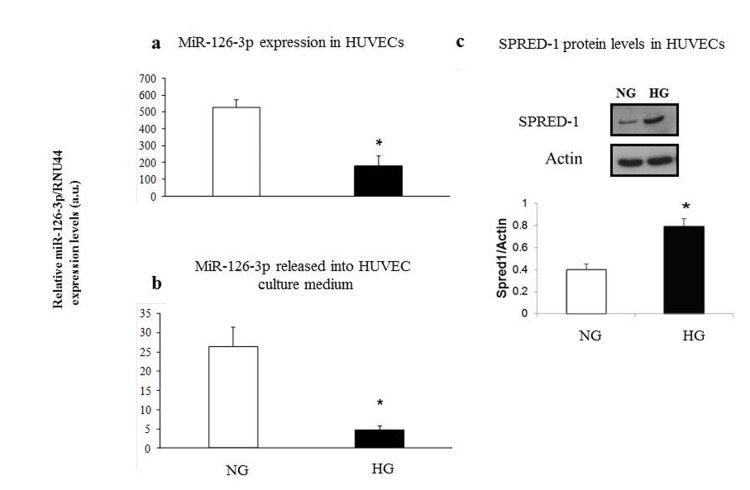
Relative miR-126-3p expression and SPRED1-protein levels in intermediate age HUVECs undergoing senescence in normoglyacemic and hyperglycemic conditions Intracellular miR-126-3p and miR-126-3p recovered from medium in normoglycemic (NG) (**a**) and hyperglycemic (HG) cultures (**b**). SPRED1-protein levels in normoglycemic (NG) and hyperglycemic (HG) cultures (**c**). Hyperglycemic medium contained 25 mM glucose. Mannitol was used as an osmotic control in the normoglycemic cultures. MiR-126-3p is expressed as 2^ΔΔΔCt^ normalized with RNU44. * GLM, p<0.05.

We found significantly decreased intra- and extracellular miR-126-3p levels in HUVECs cultured in high-glucose medium (intracellular miR-126-3p: high- vs. low-glucose medium: 173 ± 63 vs. 520 ± 38, p<0.05; extracellular miR-126-3p: high- vs. low-glucose medium: 4.7 ± 1.3 vs. 25.3 ± 7.4, p<0.05) (Fig. 6a and 6b). A significant increase in SPRED1 protein levels was observed in intermediate-age HUVECs cultured under hyperglycemic condition (HG) vs. cells cultured in normoglycaemia (NG) (Fig. 6c).

## DISCUSSION

To the best of our knowledge, this is the first study documenting a significant age-related increase in plasma miR-126-3p levels in healthy subjects. In a previous profiling analysis of plasma miRs from healthy subjects of different ages we found a non-monotonic age-related trend for miR-21 and miR-126-3p, both of which were up-regulated in octogenarians compared with subjects in their twenties [27]. In the present study the increase in plasma miR-126-3p was demonstrated in 136 healthy CTR subjects aged 20 to 90 years. Since several studies showed that miR-126-3p is expressed primarily by ECs, EPCs and platelets [37, 38, 43] and since it is difficult to study the senescence state of ECs *in vivo* both as progenitors and as differentiated ECs, we investigated age-related changes of miR-126-3p synthesis and release in senescent HUVECs taken as a model of human ECs [34].

Microarray analysis of miRs expression during replicative senescence of HUVEC did not unraveled miR-126-3p significant deregulation. However, RT-PCR based quantification allowed us to identify significant differences in miR-126-3p expression in HUVECs during replicative senescence [44, 45].

Interestingly, miR-126-3p synthesis and release were both significantly increased in senescent compared with younger cells, confirming the *in vivo* findings. These are the first data documenting increased miR-126-3p release in senescent compared with younger cells, and suggest that miR-126-3p could be an active component of the SASP. An intriguing hypothesis is that specific miRs, including miR-126-3p, could be released by senescent cells as active SASP components, stimulating or reducing the effect, on neighboring cells, of the pro-inflammatory molecules released by senescent cells. Notably, Rippe and coauthors showed reduced miR-126-3p expression in human aortic endothelial cells (HAECs) characterized by a senescent phenotype [46]. The differential expression of miR-126-3p during cellular senescence observed in different cell types may be due to the different metabolic set-up of cells, to different *in vitro* culture conditions, and/or to a different standardization of expression values. MiR-126-3p is considered as a master regulator of physiological angiogenesis, enhancing production of anti-inflammatory chemokines and promoting EPC recruitment, supposedly as a protective mechanism promoting tissue homeostasis [32, 42]. It has previously been demonstrated that increased miR-126-3p levels promote vascular endothelial growth factor (VEGF) signaling and have a positive effect on vascular protection [42, 47]. Interestingly SPRED-1 protein, a validated miR-126-3p target [36], is down-regulated in senescent compared with young cells. MiR-126-3p over-expression enhances cell survival through reduction of SPRED-1 and consequent activation of Ras/ERK/VEGF and PI3K/Akt/eNOS signaling pathways, which are involved in promoting cell differentiation and survival [49, 50]. It may thus be hypothesized that survival signal activation via SPRED-1 down-regulation may be one of the mechanisms by which loss of replicative and survival capacity is at least partly offset by cells undergoing replicative senescence. Increased levels of circulating miR-126-3p during aging are therefore likely to constitute a positive compensatory mechanism to reduce cell dysfunctions occurring during normal aging.

It has recently been demonstrated that exosomes in the bloodstream derive from different cell types, mainly platelets [51]. However, the release of exosome-like microvesicles has been reported to increase during replicative senescence, a phenomenon that may considerably change the contribution of each cell type to circulating miRNAs *in vivo* [51]. The contribution of ECs to plasma miR-126-3p levels could significantly increase during aging as a consequence of the accumulation of different proportions of functional/ dysfunctional senescent ECs in aged individuals according to their health status.

Interestingly, plasma miR-126-3p was significantly lower in T2DM patients, especially in those with poor glycemic control, than in age-matched CTR subjects. A significant reduction in intracellular miR-126-3p and in miR-126-3p released into the culture medium was also observed in HUVECs undergoing senescence cultured in hyperglycemic medium. Notably, plasma miR-126-3p of T2DM patients showed no age-related trends or significant correlations with platelet count. These data strongly suggest that the hyperglycemia associated with diabetes can offset the survival-enhancing strategies enacted by cells, both ECs and platelets, undergoing senescence. Our findings agree with recent data showing a significant reduction in miR-126-3p released by HUVECs cultured in hyperglycemic medium compared with HUVECs cultured in normoglycemic medium [22]. Moreover, EPCs from diabetic patients show lower miR-126-3p expression compared with EPCs from healthy subjects [40]. Given that hyperglycemia negatively affects EC function and increases oxidative stress and inflammation in subjects with diabetes [52], the finding that miR-126-3p levels were lower in cells and plasma from T2DM patients is not surprising. Notably, miR-126-3p expression seems to be deregulated, specifically, down-regulated, in various age-related disorders, suggesting that the compensatory mechanisms enacted by senescent/dys-functional cells could be blunted in age-related conditions sharing an unbalanced pro-inflammatory background [53].

With regard to the mechanism by which hyperglycemia modulates miR expression, increasing evidence suggest that glucose concentration in medium can modulate miRs expression in vitro, and glycaemia levels can modulate miRs expression in vivo, but the exact mechanism has not been described [54, 55]. Recent findings provide insights into the glycemia-related modulation of miRNAs through signaling-mediated changes in transcription factor activity and epigenetic histone acetylation [56].

MiR-126 is encoded by intron 7 of the EGF-like domain 7 (EGFL7) gene, also known as Vascular Endothelial-statin (VE-statin) which is under the transcriptional control of the E-twenty six family of transcription factors (Ets-1 and -2) [57]. In the resting endothelium, ETS1 is expressed at a very low level. During angiogenesis or re-endothelialisation, ETS1 is transiently expressed at high levels in endothelial cells, suggesting that during the process of vessel formation or repair, upregulation of ETS1 transcription factor expression is required [58]. During replicative senescence an increased expression of ETS1 could induce the increasing of miR-126 expression. Notably, diabetes leads to dysregulated activation of Ets, which blocks the functional activity of progenitor cells and their commitment towards the endothelial cell lineage, even if the molecular mechanisms underlying the reduced endothelial progenitor cell number and function by high glucose are not yet clearly defined. Interestingly, one of the main targets of mir-126 is the host gene EGFL7, which regulates the proper spatial organization of ECs within each sprout and influences their collective movement.

Notably, ApoAI levels significantly correlated with plasma miR-126-3p in our T2DM patients, because miR-126-3p, like other circulating miRs, has been detected in HDL and, to a lesser extent, LDL [59]. The finding prompted us to test whether drugs that modify ApoAI may affect plasma miR-126-3p levels. To do this, plasma miR-126-3p was compared in T2DM subjects taking and not taking statins and was found to be significantly reduced in the latter subjects. These data agree with reports showing that atorvastatin selectively impacts miR-126-3p expression in EPCs from patients with cardiovascular disease [60, 61]. Notably, other pharmacological treatments, e.g. aspirin, have been documented to interfere with inter- and intracellular miR-126-3p expression, with effects likely depending on cell type-specific functions and targets [33, 40, 41, 62].

In conclusion, circulating miR-126-3p can be considered as a biomarker of physiological endothelial senescence in normoglycemic elderly/old subjects and appears to be involved in a mechanism that may be disrupted in aged diabetic patients. Our findings reinforce the hypothesis that exposure to high glucose levels may induce accumulation of dysfunctional endothelial senescent cells and/or impair their survival strategy in T2DM patients, thus increasing the risk of developing micro and macrovascular complications.

## MATERIALS AND METHODS

### Study population

Participants were recruited from the Italian National Research Center on Aging (INRCA), Ancona. All subjects gave their written informed consent to participate in the study, which was approved by INRCA's Ethics Committee.

Plasma miR-126-3p was measured in 329 subjects who included 193 patients with T2DM aged 40-80 years (M=109, F=84) and 136 healthy control subjects (CTR) aged 20-90 years (M=66, F=70). To ensure perfect matching, a subset of 92 individuals aged 40-80 years with the same mean age and M/F proportion as the group of T2DM patients was selected from the CTR group and used for some comparisons.

The health status of CTR subjects was assessed using standardized questionnaires, laboratory assays and physical examination. Subjects were considered healthy if at the time of blood collection they did not have any major acute and/or chronic age-related disease such as AMI, CHF, Alzheimer's disease (AD), T2DM or cancer. Subjects with a Cumulative Illness Rating Scale (CIRS) > 2, which indicates a comorbid state, were excluded [63].

Inclusion criteria for T2DM patients were: a diagnosis of T2DM according to American Diabetes Association Criteria [64], a body mass index (BMI) < 40 kg/m^2^, and an ability and willingness to provide written informed consent and to comply with study requirements. Information collected included vital signs, anthropometric data, and medical history.

### Total RNA extraction from plasma

Peripheral blood samples were collected in EDTA-coated tubes (Venoject, Terumo Europe NV, Leuven, Belgium). After two subsequent spins, total RNA was extracted from 100 Δl of plasma using an RNA purification kit (Norgen Biotek Corporation, Thorold, ON, Canada) that isolates enriched miR species. RNA was stored at -80 °C until use. The synthetic *Caenorhabditis elegans* miR, cel-miR-39, was spiked into human plasma before RNA extraction. Only samples with cel-miR-39 recovery > 95 % were used in subsequent analyses.

### HUVEC culture

HUVECs were purchased from Clonetics Corporation (Lonza, Basel, Switzerland) and cultured in EGM-2endothelial growth medium (Lonza). Three different samples of HUVECs derived from pools of donors were used. Briefly, fresh cells were seeded at a density of 2,500 cells/cm^2^ in T 75 flasks, the medium was changed every 48 h. Cultures reached confluence after 6-7 days, as assessed by light microscopic examination, and were passaged at weekly intervals. After trypsinization and before replating, harvested cells were counted using a hemocytometer. Replicative senescence was studied by culturing cells up to the 12^th^ passage, as described previously [65]. Viable cells were counted at each passage by trypan blue staining; population doublings (PDs) were determined as current PDs=last PDs+log2 (collected cell number / seeded cell number); cumulative population doubling (CPD) was calculated as the sum of all PD changes, as recently described by our group [45]. Cells were divided into young (CPD=10 ± 2), intermediate age (CPD=30 ± 2), and senescent (CPD=40 ± 2).

SA-β-gal activity was assessed as described previously [45].

Intermediate-age HUVECs were cultured in high glucose (25 mM) medium—a hyperglycemia-like environment—until they reached replicative senescence.

### Total RNA extraction from HUVECs and culture medium

MiRs were purified from HUVECs and from 100 Δl of culture medium using the same RNA purification kit used for plasma samples (Norgen Biotek).

### Protein extraction and immunoblotting

Cells were washed twice in cold PBS. Total protein was extracted using RIPA buffer (150 mMNaCl, 10 mM Tris, pH 7.2, 0.1 % SDS, 1.0 % Triton X-100, 5 mM EDTA, pH 8.0) containing a protease inhibitor cocktail (Roche Applied Science, Indianapolis, IN). Protein concentration was determined using Bradford Reagent (Sigma-Aldrich, Milano, Italy). Total protein extracts (40 μg) were separated by 10 % SDS-PAGE and transferred to PVDF membranes (Bio-Rad, Hercules, CA). Membranes were incubated overnight with primary anti-SPRED-1 antibody (Thermo Scientific, Pierce Biotechnology, Rockford, IL) and subsequently incubated with a secondary antibody conjugated to horseradish peroxidase for 1 h at room temperature. Immuno-reactive proteins were visualized using ECL Plus chemiluminescence substrate (GE Healthcare, Pittsburgh, PA). Membranes were incubated with anti β-actin (Santa Cruz Biotechnology, Santa Cruz, CA) as an endogenous control.

### MicroRNA quantification by RT q-PCR

MiR expression was quantified using a modified real-time approach with the TaqMan miRNA reverse transcription kit and a miRNA assay (Applied Biosystems, Foster City, CA). Briefly, total RNA was reverse transcribed with a TaqMan MicroRNA RT kit. The 5 μl of reverse transcription (RT) reactions contained 1 μl of each miR-specific stem-loop primer, 1.7 μl of input RNA, 0.4 μl of 10 mM dNTPs, 0.3 μl reverse transcriptase, 0.5 μl 10X buffer, 0.6 μl RNAse inhibitor diluted 1:10, and 0.5 μl H_2_O. The mixture was incubated at 16 °C for 30 min, at 42 °C for 30 min and at 85 °C for 5 min. Quantitative real-time PCR was subsequently performed. The 5 μl PCR reaction included 0.25 μl 20x TaqMan MicroRNA Assay, which contained the PCR primers and probes (5'-FAM), 2.75 μl 2x TaqMan Universal Master mix no UNG (Applied Biosystems) and 2.00 μl RT product. The reaction was first incubated at 95 °C for 2 min, followed by 40 cycles of 95 °C for 15 sec and 60 °C for 1 min. Data were analyzed with Real Time PCR Opticon Monitor version 2 (MJ Research, Bio-Rad) with automatic Ct setting for adjusting the baseline and threshold for Ct determination.

To date, very few studies have measured standardized reference miRNA levels in plasma or serum [66, 67]. Some have demonstrated that the endogenous non-miRNA controls used in miRNA expression studies, such as RNU6B, RNU44 and RNU48, are degraded in serum. To obtain accurate and reproducible results, the relative expression of miRs was therefore quantified using synthetic *C. elegans* miRNA (cel-miR-39) as a spiked in control for RNA extraction and as the reference miR.

MiR expression in HUVECs and miR release in the culture medium were evaluated using RNU44 as the reference. Each reaction was performed in duplicate.

### Measurement of telomere length

High molecular weight DNA was isolated from white blood cells. Telomere length was measured as abundance of telomeric template (T) vs. a single gene copy (S) by quantitative real-time PCR as described previously [68].

### p16INK4a mRNA expression

For p16 mRNA gene expression, total RNA was reverse transcribed using RT^2^ First Strand Kit (Norgen Biotek) according to the manufacturer's instructions. The cDNA thus obtained was used for subsequent quantitative real time PCR (qPCR). qPCR reactions were conducted on a MyiQ Single Color Real-Time PCR Detection System (Bio-Rad) in a 15 μl total reaction volume using iQ^TM^ SYBR Green Supermix (Bio-Rad). The mRNA expression of p16 was calculated with reference to three reference genes (β-actin, β2M and HPRT1). Primer concentration varied with the primer used: ß-actin and HPRT1 were used at 300 nM, the others at 400 nM concentration. Each reaction was run in duplicate and always included a no-template control.

The primers for p16 (ID 8207) were taken from published and experimentally validated assays in RT Primer DB, a freely accessible database (http://www.rtprimerdb.org) [69]; the other primers were as described previously [70].

The qPCR reaction was programmed to start with a 3 min denaturation step at 95 °C for polymerase activation followed by 40 cycles of 15 sec denaturation at 95 °C and 30 sec of annealing/extension at 60 °C, during which fluorescence was measured. Next, a melting curve was constructed by raising the temperature from 55 °C to 95 °C in sequential 0.5 °C steps for 6 sec. PCR efficiencies were all between 90 % and 110 % and were taken into account when calculating Ct values. mRNA quantification was assessed using the 2^−ΔΔCt^ method.

### Laboratory assays

White blood cell, monocyte and platelet counts were performed by standard automated procedures (Sysmex XE-2100, Kobe, Japan). Blood concentrations of HbA1c, fasting insulin, fibrinogen, and apolipoprotein A-I and B (ApoAI and ApoB) were measured by standard procedures. An immunoenzymatic method (Biopool, Sweden) was used for PAI-1 antigen. Total and HDL cholesterol, triglycerides, creatinine, and fasting glucose were measured by an enzymatic colorimetric test on automated clinical chemistry analyzers (Roche-Hitachi, Basel, Switzerland). Highly sensitive C-reactive protein (hsCRP) was determined by the particle-enhanced immunoturbidimetric assay (CRP High Sensitive, Roche-Hitachi). Unless otherwise specified, the same blood sample was used for miRNA analysis and for determination of the other biomarkers.

### Statistical methods

The expression levels of circulating miRs are reported as relative expression compared with cel miR-39. The relative expression of each miR was reported as 2^−ΔΔCt^.

Continuous variables are presented as mean ± standard deviation (SD), categorical variables as relative frequencies. The independent sample t-test was used to compare continuous variables, the chi-square test to compare relative frequencies.

Correlations between parameters were calculated using Pearson correlation coefficient or partial correlation coefficient (R) controlled for age.

Mean standardized values of plasma miR-126-3p levels in T2DM and CTR subjects were calculated using the standard procedure: standard measure= X- mean/SD. Among groups comparisons were conducted with General Linear Models (GLM) adjusted for age and gender, with Bonferroni's correction when more than two groups were compared, and with analysis of variance (ANOVA) or covariance (ANCOVA) as appropriate. All experiments were performed at least in triplicate.

Data analysis was carried out with the SPSS/Win program version 20 (SPSS, Chicago, IL). Statistical significance was defined as a two-tailed p value < 0.05.

## SUPPLEMENTARY TABLE


