# Supplementary material for: Age- and glycemia-related miR-126-3p levels in plasma and endothelial cells
Source: Aging (Albany NY). 2014 Oct 7;6(9):771–86. doi: 10.18632/aging.100693 (PMC4221921; doi:10.18632/aging.100693)
Supplement: Supplementary file 1 [file aging-06-771-s001.pdf]

SUPPLEMENTARY TABLE

Supplementary Table 1. Circulating miR-126 and biological variables: partial correlation coefficient controlled for age

Plasma miR-126	Partial R coefficient	P
**Fasting glucose**	-0.15	0.02
**HbA1c**	-0.15	0.01
**Platelets**	0.17	0.005
**ApoAI**	0.15	0.03

Only significant results are shown considering a correlation coefficient cut-off of 0.15 and a p value <0.05.
